# Guideline-Based Critical Care Pathway Improves Long-Term Clinical Outcomes in Patients with Acute Coronary Syndrome

**DOI:** 10.1038/s41598-019-53348-2

**Published:** 2019-11-14

**Authors:** Jo-Jo Hai, Chun-Ka Wong, Ka-Chun Un, Ka-Lam Wong, Zhe-Yu Zhang, Pak-Hei Chan, Yui-Ming Lam, Wing-Sze Chan, Cheung-Chi Lam, Chor-Cheung Tam, Yiu-Tung Wong, See-Yue Yung, Ki-Wan Chan, Chung-Wah Siu, Chu-Pak Lau, Hung-Fat Tse

**Affiliations:** 1 Division of Cardiology, Department of Medicine, Queen Mary Hospital, the University of Hong Kong, Hong Kong, China; 2 Division of Cardiology, Department of Medicine, the University of Hong Kong Shenzhen Hospital, Shenzhen, China; 3 Shenzhen Institutes of Research and Innovation, University of Hong Kong, Shenzhen, China; 4 Hong Kong-Guangdong Joint Laboratory on Stem Cell and Regenerative Medicine, the University of Hong Kong, Hong Kong, China

**Keywords:** Cardiology, Myocardial infarction

## Abstract

Implementation of a critical care pathway (CCP) for acute coronary syndrome (ACS) has been shown to improve early compliance to guideline-directed therapies and reduce early mortality. Nevertheless its long-term impact on the compliance with medications or clinical outcomes remains unknown. Between 2004 and 2015, 2023 consecutive patients were admitted to our coronary care unit with ACS. We retrospectively compared the outcomes of 628 versus 1059 patients (mean age 66.1 ± 13.3 years, 74% male) managed before and after full implementation of a CCP. Compared with standard care, implementation of the CCP significantly increased coronary revascularization and long-term compliance with guideline-directed medical therapy (both P < 0.01). After a mean follow-up of 66.5 ± 44.0 months, 46.7% and 22.2% patients admitted before and after implementation of the CCP, respectively, died. Kaplan-Meier analyses showed that patients managed by CCP had better overall survival (P = 0.03) than those managed with standard care. After adjustment for clinical covariates and coronary anatomy, CCP remained independently predictive of better survival from all-cause mortality [hazard ratio (HR): 0.75, 95%confidence intervals (CI): 0.62–0.92, P < 0.01]. Stepwise multivariate cox regression model showed that both revascularization (HR: 0.55, 95%CI: 0.45–0.68, P < 0.01) and compliance to statin (HR: 0.70, 95%CI: 0.58–0.85, P < 0.01) were accountable for the improved outcome.

## Introduction

The management of acute coronary syndrome (ACS) has evolved rapidly over the past few decades. Timely revascularization and optimal medical therapy including dual antiplatelet therapy, statins, betablockers and angiotensinogen-converting enzyme inhibitors/angiotensin II receptor blockers (ACEI/ARB), have all been shown to improve clinical outcomes^1–5^. Despite being incorporated into clinical guidelines^1,2,4,5^, compliance with evidence-based medical therapy remains suboptimal, especially in Asian populations^6–19^. Implementation of a critical care pathway (CCP), which encompasses standardized management orders, in-patient education and performance feedback, has been shown to enhance appropriate in-hospital and post-discharge care for patients admitted with ACS^20–22^. Importantly, this improved compliance with guideline-directed therapies has been shown to be associated with a reduced 30-day and one-year mortality^21,23,24^. Indeed our previous study showed that implementation of a CCP for ACS improved adherence to evidence-based therapies during hospitalization and prescription of guideline-recommended medications at discharge, which was translated into a reduced 30-day and 6-month all-cause mortality^25^. Nevertheless whether CCP enhances compliance to guideline-directed medical therapy or improves clinical outcomes in the long-term remains unclear. In this study, we further investigated the impact of a CCP on long-term compliance to guideline-directed medical therapy, and subsequent cardiovascular outcomes in patients admitted with ACS.

## Methods

### Study design

This study was approved by the local Institutional Review Board and carried out in accordance with good clinical practice guidelines. Informed consent was waived by the Institutional Review Board due to retrospective nature of this study. Consecutive patients who were admitted into the coronary care unit of Queen Mary Hospital, Hong Kong, for ACS and survived to hospital discharge were included in this study^26,27^. Those who had incomplete medical records at the index hospitalization or were lost to follow-up were excluded from analysis.

Our institution introduced the CCP for ACS at the end of 2007 (supplementary materials). In short, the CCP for ACS encompasses a list of standardized management orders aiming at achieving 1) early recognition of ACS; 2) timely reperfusion; 3) prompt initiation of guideline-recommended medical therapy; 4) early revascularization; 5) early patient education; 6) improved utilization of cardiac rehabilitation and smoking cessation services. After the initial run-in phase for staff training and consolidation of standard procedures, the pathway was fully implemented in the current form in 2009. As a result, we divided patients into those who were admitted prior to the introduction (i.e. pre-CCP; 2004–2007) and after full implementation of the CCP for ACS (i.e. post-CCP; 2009–2015) for analysis. Their baseline demographic features and clinical courses and treatment during the index hospitalization were retrieved. Echocardiographic measurement of left ventricular ejection fraction (LVEF) performed one month after ACS was used in this study^28^, except in patients who died within the first 30 days. Coronary stenosis ≥ 50% at the left main coronary artery or ≥70% at a non-left main coronary artery, or a coronary lesion with fractional flow reserve ≤ 0.80, was considered significant^29^. Successful revascularization was defined as successful restoration of anterograde flow in culprit coronary lesions. Prior coronary artery disease was defined as the presence of a significant coronary lesion diagnosed by conventional coronary angiography or computed tomography, or prior myocardial infarction. Chronic kidney disease was defined as an estimated glomerular filtration rate < 60 ml/min/1.73 m^2^ by the Modification of Diet in Renal Disease formula^30^. Clinical data for subsequent hospital admissions, emergency department visits, outpatient clinic follow-ups and death were recorded from the comprehensive electronic medical system of the hospital.

Use of guideline-directed medical therapy, including clopidogrel, statin, betablocker and ACEI/ARB, were assessed on discharge and at all subsequent clinical visits. Long-term compliance to a guideline-directed medical therapy was defined prescription of a medication at discharge and follow-up visits with a medication possession ratio (i.e. the proportion of patients’ time on the drug) ≥ 80% at one year for clopidogrel, and at the last follow-up visit for statin, betablocker and ACEI/ARB^19,31^. Causes of death were classified according to the Hinkle-Thaler Scheme, except that patients with documented non-arrhythmic sudden death by implantable cardioverter defibrillator or cardiac monitor at the time of cardiac arrest were excluded from sudden arrhythmic death^32,33^. The primary endpoint of this study was all-cause mortality. The secondary outcomes of interest were cardiovascular death and sudden arrhythmic death/sustained ventricular tachyarrhythmias requiring intervention by medical personnel or appropriate implantable cardioverter defibrillator therapy.

### Statistical analysis

Parametric and non-parametric continuous variables are expressed as mean [standard deviation (SD)] and median [interquartile range (IQR)], respectively. Categorical variables are presented in frequency tables. Continuous and categorical variables were compared using Student’s *t* test, Mann-Whitney U test or Fisher’s exact test, as appropriate. To determine whether patients had reduced all-cause mortality, cardiovascular mortality or sudden arrhythmic death or sustained ventricular tachyarrhythmias requiring intervention after implementation of a CCP, survival curves were plotted by the Kaplan-Meier method and compared using log-rank tests. To eliminate population selection bias, multivariate Cox regression analysis was performed to adjust for baseline clinical characteristics and coronary anatomy was performed to obtain the hazard ratio (HR) and 95% confidence intervals (CI) of implementation of a CCP to predict clinical outcomes. To determine clinical covariates and management strategies that were associated with improved all-cause mortality, a multivariate Cox regression model was built using stepwise forward selection that maximized the likelihood estimates. Non-parametric continuous variables were log-transformed, and proportional assumption was verified using graphical methods. Calculations were performed using SPSS software (version 24.0). A 2-sided *P*-value < 0.05 was considered statistically significant.

## Results

Overall, 2128 patients admitted into our coronary care unit with ACS between 2004 and 2015. Among them, 95 (4.5%) who had incomplete medical records at the index admission were excluded. Of the 2033 patients who were identified, 705 (34.7%) were admitted during the pre-CCP period, 188 (9.2%) were admitted during the run-in period and 1140 (56.1%) were admitted during the post-CCP period. A total of 81 (7.1%) patients during the pre-CCP period and 77 (10.9%) patients during the post-CCP period died in hospital. As a result, 628 patients admitted during the pre-CCP period and 1059 patients admitted during the post-CCP period who survived to hospital discharge were included in the final analysis.

Their mean age was 66.1 ± 13.3 years and 1254 (74.3%) were male. The clinical characteristics, and treatment of patients admitted before and after implementation of the CCP for ACS are summarized in Table 1. Patients admitted after implementation of the CCP shared similar baseline characteristics with those admitted before implementation of the CCP, except that more patients in the former group had a history of hypertension and fewer patients had chronic kidney disease (both *P* < 0.01). The proportion of patients who underwent successful revascularization was significantly higher in the post- than the pre-CCP period, and a higher proportion of them were treated with non-bare metal stents (both *P* < 0.01). Importantly, the peak creatine kinase was lower (*P* = 0.02) and fewer patients had a LVEF ≤ 35% one month after ACS (*P* < 0.01) after implementation of CCP. Nevertheless, the proportion of patients who had implantable cardioverter defibrillator or those who participated in our cardiac rehabilitation program were similar.

**Table 1:** Clinical characteristics, coronary anatomy and treatment of patients who were discharged with ACS before and after implementation of CCP.

Implementation of CCP	Before (N = 628)	After (N = 1059)	*P-*value
Age, years	66.8 ± 13.0	65.6 ± 13.5	0.08
Male, n (%)	457 (72.8)	797 (75.3)	0.27
ST elevated myocardial infarction, n (%)	397 (63.2)	688 (65.0)*	0.49
Killip class			<0.01
I, n (%)	418 (66.6)	586 (55.3)	
II, n (%)	99 (15.8)	288 (27.2)	
III, n (%)	81 (12.9)	139 (13.1)	
IV, n (%)	30 (4.8)	46 (4.3)	
Smoker, n (%)	314 (50.0)	482 (45.5)	0.08
Past medical history			
Hypertension, n (%)	308 (49.0)	596 (56.3)	<0.01
Diabetes mellitus, n (%)	269 (42.8)	406 (38.3)	0.07
Hyperlipidemia, n (%)	263 (41.9)	439 (41.5)	0.88
ACS, n (%)	27 (4.3)	46 (4.3)	1.00
Chronic kidney disease, n (%)	256 (40.8)	340 (32.1)	<0.01
Baseline LDL-C (mmol/L)	2.90 ± 1.00	2.82 ± 1.32	0.16
LVEF at one month			<0.01
≥50%, n (%)	282 (44.9)	440 (41.5)	
36–49%, n (%)	183 (29.1)	402 (38.0)	
≤35%, n (%)	163 (26.0)	217 (20.5)	
Creatine kinase (IU/L)	1248 (2351)	1145 (2224)	0.02
Coronary angiography			
Left main disease, n (%)	34 (5.4)	52 (4.9)	0.65
Triple vessel disease, n (%)	230 (36.6)	388 (36.6)	1.00
Revascularization, n (%)	468 (74.5)	892 (84.2)	<0.01
Bare metal stents, n (%)	114 (18.2)	52 (4.9)	<0.01
Implantable cardioverter defibrillator, n (%)	20 (3.2)	28 (2.6)	0.55
Cardiac rehabilitation, n (%)	280 (44.6)	470 (44.4)	0.96
Long-term compliance with medications			
Clopidogrel, n (%)	399 (63.5)	955 (90.2)	<0.01
Statin, n (%)	451 (71.8)	928 (87.6)	<0.01
Betablocker, n (%)	352 (56.1)	724 (68.4)	<0.01
ACEI/ARB, n (%)	372 (59.2)	705 (66.6)	<0.01
Follow-up LDL-C (mmol/L)	2.02 ± 0.81	1.92 ± 0.80	<0.001
Follow-up LVEF			0.10
≥50%, n (%)	341 (54.3)	584 (55.1)	
36–49%, n (%)	154 (24.5)	292 (27.6)	
≤35%, n (%)	133 (21.2)	183 (17.3)	

*Among those with primary percutaneous coronary intervention performed, the median door-to-balloon time was 104 minutes, and 50.4% of them had a door-to-balloon time ≤90 minutes.

ACS: acute coronary syndrome; CCP: critical care pathway; LDL-C: low density lipoprotein-cholesterol; LVEF: left ventricular ejection fraction; ACEI/ARB: angiotensinogen-converting enzyme inhibitor/angiotensin II receptor blocker.

The mean follow-up was 49.8 ± 28.3 months for patients admitted during the post-CCP period and 94.7 ± 50.8 months for those admitted during the pre-CCP period. At the end of follow-up, compliance to clopidogrel, statin, betablocker or ACEI/ARB were higher in those who admitted after implementation of the CCP (all *P* < 0.01). Despite similar baseline low density lipoprotein-cholesterol (LDL-C), patients who admitted in the post-CCP period had a lower LDL-C than those who admitted in the pre-CCP period at follow-up (1.92 ± 0.80 vs. 2.01 ± 0.81 mmol/L, *p* < 0.01). There was also no difference in LVEF between the two groups (*p* = 0.10).

**Figure 1:**
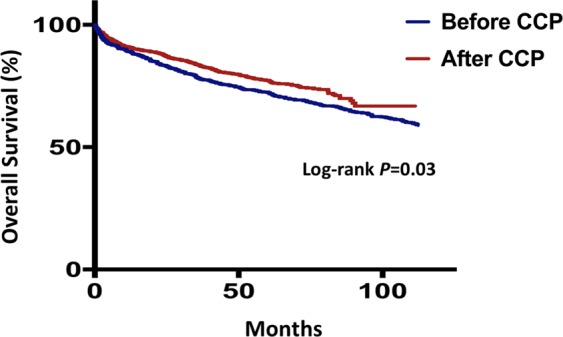
Kaplan-Meier survival curves comparing all-cause mortality between patients admitted in the pre-critical care pathway (CCP) and post-CCP periods.

**Figure 2:**
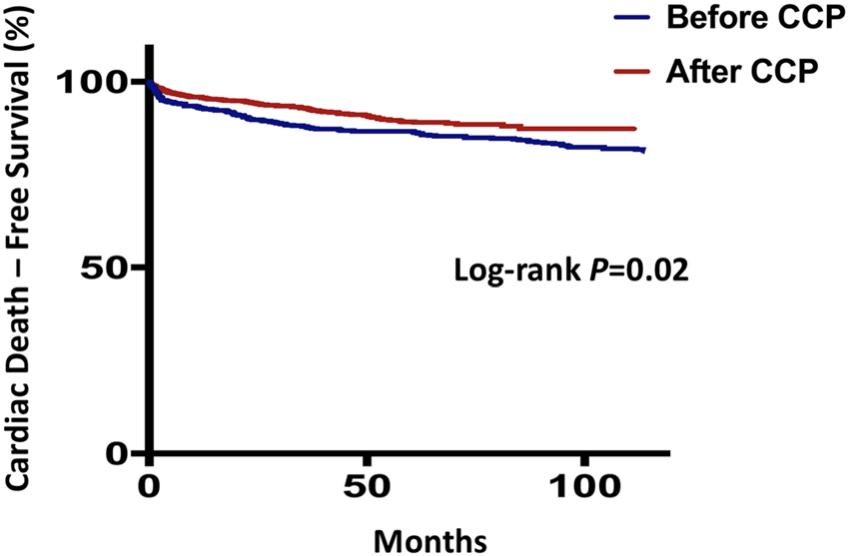
Kaplan-Meier survival curves comparing cardiac mortality between patients admitted in the pre-critical care pathway (CCP) and post-CCP periods.

**Figure 3:**
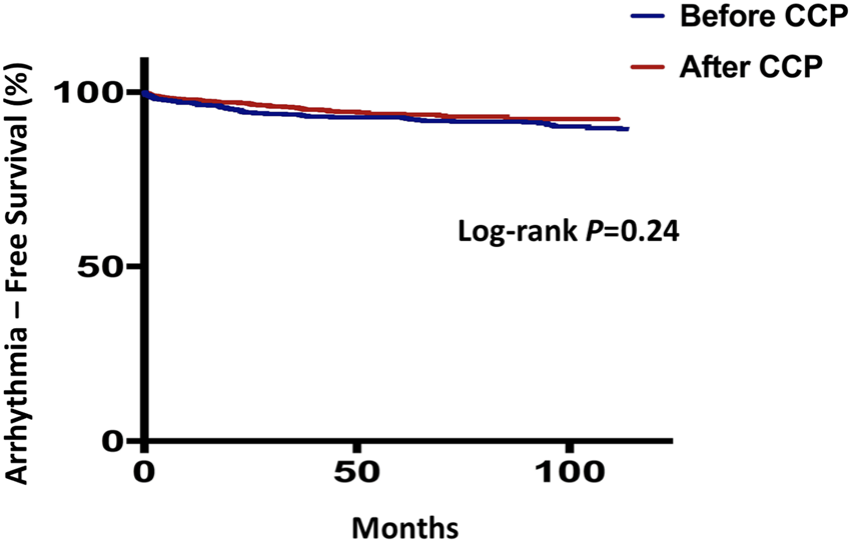
Kaplan-Meier survival curves comparing sudden arrhythmic death (SAD) or sustained ventricular tachyarrhythmias (VT) requiring intervention between patients admitted in the pre-critical care pathway (CCP) and post-CCP periods.

A total of 235 (22.2%) patients admitted after and 293 (46.7%) patients admitted before implementation of the CCP died, of whom 95 (9.0%) and 116 (18.5%) were due to cardiac causes, respectively. Sudden arrhythmic death or ventricular tachyarrhythmias requiring intervention was observed in 56 (5.3%) patients admitted after and 66 (10.5%) patients admitted before implementation of the CCP. Kaplan-Meier survival analyses showed that patients admitted after implementation of the CCP had better survival from all-cause mortality (log-rank *P* = 0.03. Fig. 1) and cardiac death (log-rank *P* = 0.02. Fig. 2), but not sudden arrhythmic death or ventricular tachyarrhythmias requiring intervention (log-rank *P* = 0.24. Fig. 3).

After adjustment for baseline characteristics of the patients, implementation of the CCP remained independently predictive of improved survival from all-cause mortality (HR: 0.75, 95% CI: 0.62–0.92, *P* < 0.01) and cardiac death (HR 0.62, 95% CI: 0.46–84, *P* < 0.01. Table 2). Clinical covariates or management strategies that might account for the improved overall survival were studied in a stepwise multivariate cox regression model. We showed that higher rate of revascularization (HR 0.55, 95% CI: 0.45–0.68, *P* < 0.01), compliance to statin (HR 0.70, 95% CI: 0.58–0.85, *P* < 0.01) and improved LDL-C at follow-up (HR 1.14, 95% CI: 1.02–1.26, *P* = 0.02) were accountable for the improved outcome (Table 3).

**Table 2:** Adjusted HR of CCP implementation to predict all-cause and cardiovascular mortality.

	All-Cause Mortality HR (95% CI)	*P-*value*	Cardiovascular Mortality HR (95% CI)	*P-*value**
Age	1.07 (1.05–1.08)	<0.01	1.04 (1.03–1.06)	<0.01
Male	1.24 (1.02–1.51)	0.03	1.13 (0.83–1.52)	0.45
ST elevated myocardial infarction	0.75 (0.62–0.91)	<0.01	0.72 (0.54–0.97)	<0.01
Killip class		<0.01		<0.01
I	Reference		Reference	
II	1.11 (0.89–1.40)	0.36	1.30 (0.90–1.87)	0.16
III	1.49 (1.18–1.87)	<0.01	1.89 (1.32–2.69)	<0.01
IV	1.16 (0.77–1.77)	0.48	1.41 (0.74–2.70)	0.30
Smoker	0.95 (0.80–1.14)	0.60	0.84 (0.64–1.12)	0.24
Past medical history				
Hypertension	1.03 (0.85–1.25)	0.77	1.40 (1.02–1.92)	0.04
Diabetes mellitus	1.27 (1.06–1.52)	0.01	1.19 (0.90–1.58)	0.22
Hyperlipidemia	0.92 (0.77–1.10)	0.34	0.96 (0.72–1.27)	0.76
ACS	1.09 (0.76–1.55)	0.66	0.90 (0.51–1.61)	0.73
Chronic kidney disease	2.18 (1.79–2.65)	<0.01	2.04 (1.49–2.78)	<0.01
Creatine kinase	0.79 (0.65–0.95)	0.02	0.77 (0.57–1.05)	0.10
LVEF		<0.01		<0.01
≥50%	Reference		Reference	
36–49%	1.26 (1.01–1.58)	0.04	1.07 (0.73–1.56)	0.75
≤35%	1.88 (1.51–2.33)	<0.01	2.36 (1.68–3.30)	<0.01
Coronary angiography				
Left main disease	0.73 (0.50–1.06)	0.10	0.84 (0.49–1.46)	0.54
Triple vessel disease	0.67 (0.54–0.84)	<0.01	0.40 (0.27–0.60)	<0.01
Implementation of CCP	0.75 (0.62–0.91)	<0.01	0.62 (0.46–0.84)	<0.01

*p-value for all-cause mortality.

**p-value for cardiovascular mortality.

HR: hazard ratio; CCP: critical care pathway; ACS: acute coronary syndrome; LVEF: left ventricular ejection fraction.

**Table 3:** Multivariate prediction model of all-cause mortality.

	All-Cause Mortality HR (95% CI)	*P-*value
Age	1.06 (1.05–1.07)	<0.01
Male	1.34 (1.10–1.64)	<0.01
ST elevated myocardial infarction	—	—
Killip class		0.04
I	Reference	
II	1.15 (0.91–1.44)	0.24
III	1.39 (1.11–1.75)	<0.01
IV	1.24 (0.81–1.88)	0.32
Smoker	—	—
Past medical history		
Hypertension	—	—
Diabetes mellitus	1.40 (1.18–1.67)	<0.01
Hyperlipidemia	—	—
ACS	—	—
Chronic kidney disease	2.02 (1.65–2.46)	<0.01
Creatine kinase	0.81 (0.68–0.98)	0.03
LVEF at one month		<0.01
≥50%	Reference	
36–49%	1.21 (0.95–1.55)	0.12
≤35%	1.58 (1.23–2.02)	<0.01
Coronary angiography		
Left main disease	—	—
Triple vessel disease	—	—
Revascularization	0.55 (0.45–0.68)	<0.01
Implantable cardioverter defibrillator	—	—
Cardiac rehabilitation	0.66 (0.54–0.82)	<0.01
Bare metal stents	—	—
Long-term compliance with medications		
Clopidogrel	—	—
Statin	0.70 (0.58–0.85)	<0.01
Betablocker	—	—
ACEI/ARB	—	—
Follow-up LDL-C (mmol/L)	1.14 (1.02–1.26)	0.02
Follow up LVEF		0.01
≥50%, n (%)	Reference	
36–49%, n (%)	0.99 (0.78–1.25)	0.90
≤35%, n (%)	1.39 (1.09–1.77)	<0.01

ACS: acute coronary syndrome; LVEF: left ventricular ejection fraction; ACEI/ARB: angiotensinogen-converting enzyme inhibitor/angiotensin II receptor blocker; LDL-C: low density lipoprotein-cholesterol.

## Discussion

Our previous study showed that implementation of a CCP for ACS significantly enhances the use of guideline-directed therapies during hospitalization and at discharge, as well as improved early survival, in an Asian population^25^. Here, we extended this evaluation to long-term follow-up, and have several important findings. First, implementation of the CCP for ACS was not only associated with improved prescription of guideline-directed medical therapy at discharge, but also reduced discontinuation of these medications at follow-up. Second, the benefit of the CCP was not limited to suburban or non-tertiary institutions with low adherence to clinical guidelines, but also applied to a coronary care unit of a major academic institution with an averaged baseline adherence to evidence-based therapies. Third, implementation of the CCP for ACS did not only improve in-hospital and early mortality, but also long-term survival from all-cause and cardiac mortality. Importantly, this improvement in long-term outcome was independent of patients’ clinical characteristics and coronary anatomy, thus eliminating a population selection bias. Last, patients admitted after implementation of the CCP were significantly more likely to undergo successful revascularization and comply with long-term statin, both of which were shown to be associated with better overall survival.

Although advances in acute management as well as secondary prevention therapies have markedly reduced the mortality and morbidity associated with ACS in high-income western countries^1–5^, compliance with evidence-based therapies by physicians and patients in many other regions, including Asia, remains suboptimal^6–17^. In India, as few as 48% of patients admitted for ST elevated myocardial infarction received any form of reperfusion therapy, while the proportion of ACS patients prescribed P_2_Y_12_ inhibitor, statin, betablocker or ACEI/ARB on discharge was 79.4%, 70.1%, 62.7% and 25.5%, respectively^17^. In Malaysia, the prescription rates of dual antiplatelet therapy, betablocker and ACEI/ARB on discharge were 82%, 80.3% and 69.7%, respectively^16^. Even in a higher-income country such as Japan and Korea, prescription rates of statin, betablocker and ACEI/ARB remained in the range of 40–80%^14,18,19^. A multicenter study conducted in China further showed that the compliance to evidence-based therapies reduce over time. Although 44.6%, 80.4%, 70.0% and 75.7% of all ACS patients were prescribed P_2_Y_12_ inhibitor, statin, betablocker and ACEI/ARB on discharge, respectively, only 19.4%, 59.4%, 69.9% and 67.9% remained on these medications at one year^13^.

There are multiple reasons for non-compliance with evidence-based therapies. Physician awareness, cultural beliefs, patient education, financial affordability and accessibility to medical facilities are all implicated. Although there is no straightforward solution to our current situation, experiences from Western countries have shown that implementation of a CCP for ACS significantly improves compliance with guideline-recommended therapies as well as overall survival for up to one year^20–22^. The results of this study further showed that benefit of the CCP extended to the long-term. In fact, all our patients received in-hospital assessment and education by cardiac nurses, dietitians, physiotherapists and occupational therapists as early as the second day of ACS and continued throughout the entire hospitalization period. It is likely that early education on the disease nature, its treatment, potential treatment-related side effects as well as the risk of non-compliance had imposed a positive and long-lasting impact on patients’ compliance with guideline-recommended medications.

Consistent with previous studies, we demonstrated that long-term use of statin was associated with increased all-cause mortality at follow-up^34,35^. Importantly, the benefit of long-term statin was independent to the LDL-C level at follow-up, supportive of a pleiotropic effect^36,37^. Nevertheless long-term use of betablocker or ACEI/ARB was independent predictors of late survival in this study. While early use of betablocker and ACEI/ARB has been shown to reduce infarct size, increase LVEF and improve short- and medium-term mortality^38–40^, evidence supporting their long-term use in low-risk patients with normal or near normal LVEF after ACS is scarce. Indeed, recent data have shown that continuation of betablockers beyond the first year does not reduce mortality in current era of ACS care^41^. Since most of our patients were optimally treated, as reflected by a low rate of patients with long-term LVEF ≤35%, it is likely that the benefit of long-term betablocker or ACEI/ARB is too small to be detected.

In this study, we found no differences in rates of sudden arrhythmic death or ventricular tachyarrhythmias requiring intervention following ACS after implementation of a CCP. We believe that this was due to the low rate of malignant arrhythmic events in our institution with contemporary early revascularization and optimal medical therapy^33,42^. The annualized incidence rates of sudden arrhythmic death or ventricular tachyarrhythmias were 1.35% per year prior to and 1.28% per year after implementation of the CCP. As a result, a much larger sample size is required to demonstrate any benefit of a CCP in reducing life-threatening arrhythmias.

Although our results showed that implementation of a CCP bettered acute and long-term management of ACS in our institution, there is still considerable room for improvement. The 2017 European Society of Cardiology (ESC) Guidelines for the management of ST-elevated myocardial infarction and the 2017 American Heart Association (AHA)/American College of Cardiology (ACC) Clinical Performance and Quality Measures for ACS identified comprehensive sets of indicators to assess the quality of health care and serve as a foundation for quality improvement initiatives^5,43^. These quality indicators provide objective measures of an institution’s clinical performance in service organization, reperfusion therapy, risk assessment, medical treatment and clinical outcomes. In these regards, our CCP included a pre-hospital triage, transfer and catheterization laboratory activation protocol in collaboration with other hospitals and emergency services. Furthermore, we achieved high rates of P_2_Y_12_ inhibitors and statins prescriptions, and improved utilizations of other guideline-recommended medical therapies. These accomplishments were accompanied by improved short term outcomes as described in our previous publication^25^. Nevertheless, it should be noted that our median door-to-balloon time was over 90 minutes, and only half of those who were eligible to primary percutaneous coronary intervention had a door-to-balloon time ≤ 90 minutes. As door-to-balloon time was not systematically collected in the pre-CCP period, evaluation of a trend was not feasible. However, long door-to-balloon time is often reported in other Asian countries^44^, which is suggestive of structural reasons in our region for the delayed reperfusion that might not be able to be tackled by our current CCP. For examples, previous studies have shown delayed reperfusion could be caused by atypical symptoms or electrocardiographic changes, concomitant respiratory failure or cardiac arrest requiring urgent management prior to transferral to catheterization laboratory, as well as presentation to medical facilities at off-work hours^44–46^. In addition, time spent on obtaining informed consent has been consistently shown to be an important cause of delayed reperfusion in the Asian populations^44,47,48^. Regrettably, the door-to-balloon time itself does not provide detail insights required to analyse reasons for the delay. In 2017, the ESC Guidelines for ST-elevated myocardial infarction and the AHA/ACC Clinical Performance Measures for ACS proposed a ‘diagnosis-to-wire crossing time’ within 60 minutes and a ‘first medical contact-to-device time’ within 90 minutes, respectively, as new metrics to quality assessment in reperfusion therapy^5,43^. Accordingly, our institution began documentation of these new parameters in early 2018. Future evaluation of these key reperfusion times will help identify the missing links to target quality improvement.

Our study has several limitations. First, information about compliance with guideline-directed medical therapy was based the medication possession ratio whereas objective evidence such as serum drug level was lacking. Second, information about non-medical interventions that could affect clinical outcomes of our patients, such as exercise habit, was not prospectively collected. Third, reasons for non-compliance with therapies, which have important clinical implications, were not systematically evaluated. Fourth, advancement in revascularization techniques and technologies, as well as emergence of new medications including more potent statins, could not be adequately adjusted in the analysis.

To conclude, implementation of a CCP was associated with improved long-term compliance with guideline-recommended medical therapy. In particular, persistent use of statin independently predicted reduced long-term mortality. We advocate that all medical institutions consider implementation of a CCP for ACS to ensure delivery of quality care to patients to achieve the best possible long-term outcomes.
